# Patent Medicines

**Published:** 1875-01

**Authors:** M. S. Bidwell


### PATENT MEDICINES.

BY M. S. BIDWELL.

In the October number of the Bistoury, an attempt was made to show that the use of Patent Medicines, so-called, is a folly on the part of the user, and an evil to the community; and the question naturally arises—"What are you going to do about it?" In other words, how may this evil be abated? Notice the expression—it may be *abated*—it cannot be cured. So long as mankind consist of a large majority of fools, with an active minority of knaves (themselves doubtless fools in other directions) ready to prey on them, so long will fraud and humbug continue to flourish. And so long as the public demand Patent Medicines, they will be sold, and neither reason nor persuasion nor legislation will banish them. Experience shows, for instance, that a drug store cannot be successfully conducted without selling them, unless in very exceptional cases. Some years ago an Apothecaries Hall was started in New Haven, which was to be devoted to Pharmacy and the legitimate sale of drugs, nostrums being excluded, and was to be under the special patronage of the physicians. It was hoped that this patronage, with the profit on the prescription trade so far as it could be controlled by the physicians, would compensate for the loss incurred by not selling secret remedies. Nevertheless, when the writer visited that city a few years later, that same establishment was said to have the largest stock of patent Medicines in the city. The same experience has doubtless been repeated elsewhere. We anticipate, therefore, no general relief in this world—what may happen in the next is open only to conjecture. Of course these nostrums can have no chance in Heaven, being peremptorily shut out by the clause excluding "whosoever loveth or maketh a lie," as well as by the general intelligence of the population. But in the other department of the future world (omitting the consideration of any "intermediate state") they would seem likely to find a more congenial sphere; and there is nothing unreasonable in supposing that Pandemonium may have its Patent Medicine Department, probably under the supervision of Mammon, aided perhaps by Moloch, the anciant destroyer of babes, with Belial, the father of lies, as advertising agent. It is true that Milton does not mention this feature, in his description, but his silence on this point is easily explained. With all his learning, he did not know everything, and besides, he was very much fettered by classical models and poetic proprieties. Yet it does seem as if the conflict between the angels and devils would have been more natural, and perhaps have had a different result, if Satan, instead of getting up artillery and gunpowder, had equipped a few of his most persistent imps with a stock of Pills, and especially of Whisky Bitters, and sent them out to trade among his enemies. Methinks the angelic host would soon have been so demoralized that when it came to throwing mountains around, they would have been nowhere in the game.

But to come back from the future world to the present—though we cannot abolish these nostrums, yet we can, each one within the sphere of his own influence, discountenance their use, and so discharge our own duty. This each one can do, and should do as a matter of principle, by refusing to use or to recommend any secret remedy whatever. The class who probably have it in their power to do the most good in this respect are the Druggists. Physicians can do more to promote the sale of the nostrums by their recommendations; but their influence is less effective against them, because their opposition is attributed to selfish motives. But when a druggist conscientiously loses its sale, and very likely incurs the ill will of his customer besides, his course is so manifestly disinterested, and ever opposed to his own interest, that it cannot help having some weight. And when a druggist resolutely and consistently carries out this principle, which of course entails upon him a very considerable pecuniary sacrifice, ought not physicians to support him by their influence and patronage, rather than his less scrupulous rivals? As was mentioned in a former number, the most intelligent Pharmacists of the country, adopt this honest course.

But the responsibility cannot be thrown entirely on either Doctors or Druggists. Every intelligent member of the community is responsible for this evil just so far as his influence extends, and is not used against it; and if any reader shall be induced to discountenance these dangerous frauds, or strengthened in his opposition to them, the purpose of these articles will be served.

